# Critical Role of TLR4 on the Microglia Activation Induced by Maternal LPS Exposure Leading to ASD-Like Behavior of Offspring

**DOI:** 10.3389/fcell.2021.634837

**Published:** 2021-03-04

**Authors:** Lu Xiao, Junyan Yan, Di Feng, Shasha Ye, Ting Yang, Hua Wei, Tingyu Li, Wuqing Sun, Jie Chen

**Affiliations:** ^1^Chongqing Key Laboratory of Childhood Nutrition and Health, Children's Hospital of Chongqing Medical University, Chongqing, China; ^2^Ministry of Education Key Laboratory of Child Development and Disorders, National Clinical Research Center for Child Health and Disorders, Chongqing, China; ^3^Information Technological Service Center, Children's Hospital of Chongqing Medical University, Chongqing, China

**Keywords:** toll-like receptor 4, autism spectrum disorder, lipopolysaccharide, microglia, synaptic pruning

## Abstract

**Objective:** To investigate the role of TLR4 on the microglia activation in the pre-frontal cortex, which leads to autism-like behavior of the offspring induced by maternal lipopolysaccharide (LPS) exposure.

**Methods:** Pregnant TLR4^−/−^ (knockout, KO) and WT (wild type, WT) dams were intraperitoneally injected with LPS or PBS, respectively. The levels of TNFα, IL-1β, and IL-6 in the maternal serum and fetal brain were assessed with ELISA following LPS exposure. The gestation period, litter size and weight of the offspring were evaluated. Three-chamber sociability test, open field test and olfactory habituation/dishabituation test were used to assess the offspring's autism-like behavior at 7 weeks of age. Western blotting was performed to examine the levels of TLR4, Phospho-NFκB p65, IKKα, IBA-1, iNOS, Arg-1, C3, CR3A, NMDAR2A, and Syn-1 expression in the pre-frontal cortex. The morphological changes in the microglia, the distribution and expression of TLR4 were observed by immunofluorescence staining. Golgi-Cox staining was conducted to evaluate the dendritic length and spine density of the neurons in 2-week-old offspring.

**Results:** Maternal LPS stimulation increased serum TNFα and IL-6, as well as fetal brain TNFα in the WT mice. The litter size and the weight of the WT offspring were significantly reduced following maternal LPS treatment. LPS-treated WT offspring had lower social and self-exploration behavior, and greater anxiety and repetitive behaviors. The protein expression levels of TLR4 signaling pathways, including TLR4, Phospho-NFκB p65, IKKα, and IBA-1, iNOS expression were increased in the LPS-treated WT offspring, whereas Arg-1 was decreased. Maternal LPS treatment resulted in the significant reduction in the levels of the synaptic pruning-related proteins, C3 and CR3A. Moreover, the neuronal dendritic length and spine density, as well as the expression levels of the synaptic plasticity-related proteins, NMDAR2A and Syn-1 were reduced in the WT offspring; however, gestational LPS exposure had no effect on the TLR4^−/−^ offspring.

**Conclusion:** Activation of TLR4 signaling pathway following maternal LPS exposure induced the abnormal activation of microglia, which in turn was involved in excessive synaptic pruning to decrease synaptic plasticity in the offspring. This may be one of the reasons for the autism-like behavior in the offspring mice.

## Introduction

Autism spectrum disorder (ASD) represents a category of neurodevelopmental disorders characterized by social and communication impairments and restricted or repetitive behaviors; however, the precise etiology and pathophysiology remain unknown (Hyman et al., 2020). Epidemiological studies have shown that maternal infection during pregnancy may be associated with the onset of ASD in offspring (Bilbo et al., 2018). Therefore, a large number of animal studies that mimic infection during pregnancy have been established to induce an ASD-like behavior in offspring to study ASD pathogenesis (Filiano et al., 2016). Lipopolysaccharide (LPS) is a component of gram-negative bacteria and a common molecule that can imitate a maternal bacterial infection during pregnancy (Fortier et al., 2007). TLR4 is a specific LPS receptor, the specific binding of the two elements could activate the TLR4 signaling pathway and produce an inflammatory response (Lehnardt et al., 2002; Hromada-judycka, 2015; Takeda and Akira, 2015). In the central nervous system (CNS), TLR4 is primarily expressed on the microglia, which is resident in immune cells and phagocytes of the CNS (Schafer et al., 2013). Microglia immediately changes from stationary branches to phagocytic amoeboid, releasing inflammatory cytokines and chemokines, and participating in the immune response to inflammation or injury of the central nervous system (Ransohoff and Cardona, 2010). In addition, the microglia are also involved in regulating neurogenesis, myelin formation, and synaptic remodeling (Bilimoria and Stevens, 2015; Salter and Stevens, 2017); they shape neuronal connections by pruning redundant dendritic spines in early life. Moreover, inappropriate synaptic pruning is associated with several neurodegenerative diseases, including Parkinson's disease, Alzheimer's disease, and ASD (Mcdougle et al., 2015; Fernández de Cossío et al., 2017).

Studies have shown that activation of microglia through TLR4 stimulation leads to neuronal death and neuroinflammatory damage (Fernandez-lizarbe et al., 2009). Animal experiments have revealed that LPS exposure during pregnancy, leading to ASD-like behavior in offspring, as well as activation of microglia (Chen et al., 2012; Santra et al., 2016). Further studies confirmed that maternal LPS exposure is associated with abnormal synaptic pruning of the microglia in offspring with ASD-like behavior (Fernández de Cossío et al., 2017); however, the role of TLR4 in maternal LPS-induced microglia activation involved in the abnormal synaptic pruning that leads to an ASD-like behavior in offspring remains unclear.

Considering the critical role of both the microglia and TLR4 in the neuroinflammation and pathogenesis associated with many neurodegenerative disorders, our study aimed to assess whether maternal LPS exposure induces microglia activation through TLR4 stimulation and whether activation of the microglia triggers abnormal synaptic pruning leading to ASD-like behavior in offspring. Therefore, gestational WT and TLR4^−/−^ mice were exposed to LPS to induce ASD-like behavior in the offspring, respectively, and confirmed the key role of TLR4. Next, we identified the relationship between the change in TLR4 and microglia in the offspring mice. Finally, early postnatal microglia changes were involved in abnormal synaptic pruning, to verify that microglia dysfunction may be a reason for the ASD-like behavior. The present study provides novel insight into the basic mechanism through which maternal LPS induces the activation of microglia and leads to an ASD-like behavior in the offspring.

## Materials and Methods

### Animals, Grouping, and Sample Collection

This study was approved by the Animal Experimentation Ethics Committee of Chongqing Medical University (Chongqing, China) and conducted in accordance with the guidelines of the Animal Care Committee of Chongqing Medical University. TLR4^−/−^ (knockout, KO) and WT (wild type, WT) mice were obtained from the Model Animal Research Center of Nanjing University (MARC). The TLR4^−/−^ mouse strain, C57BL/10ScNJNju, was based on the C57BL/10JNju mouse strain (WT). These mice were housed in the same room with a constant airflow system, controlled temperature (22–24°C), and a 12-h light/dark cycle.

Female WT and TLR4^−/−^ mice were mated with males of the same strain overnight, and female mice with a vaginal plug observed the next morning were recorded as the 0.5 day of gestation. Treated WT dams with a single *i.p*. injection of 50 μg/kg LPS (*E. coli*; O127:B8) or an equal-volume PBS on gestational day 14.5, the offspring were termed the WT LPS group and WT PBS group, respectively. Similarly, the TLR4^−/−^ dams received an intraperitoneal injection of 50 μg/kg LPS (*E. coli*; O127:B8) or equal-volume PBS on 14.5 days of gestation. The offspring were termed as the KO LPS group and KO PBS group, respectively.

A total of 15 pregnant mice were randomly selected from each group. The maternal serum and fetal brains were collected 5 h post-intraperitoneal injection, and the remaining pregnant mice gave birth naturally. The day of birth was recorded as the 1st day after birth. Behavioral tests were performed between 49 and 56 days after birth. Part of the 2 and 7-week old pups were sacrificed, and the pre-frontal cortex of the pups were collected on ice and immediately frozen at −80°C for subsequent experiments.

### Behavioral Testing

All of the mice were tested at an age of 7 weeks, and all the behavioral tests could be conducted in a quiet and gentle environment. A test box or cage were cleaned of urine and feces, then sterilized with 75% alcohol before the next mouse was tested.

### Olfactory Habituation/Dishabituation Test

Urine was collected in advance from the adult C57 mice. The subjects were habituated in a clean, empty cage for 10 min before the experiment had been initiated. The fluid-filled swabs were suspended 10 cm from the bottom of the box to make sure the mice could smell them. Each mouse was required to sniff three types of cotton swabs containing water, mouse urine, and beer, respectively. The observation time of each cotton swab was 3 min, and the total time of the mouse sniffing cotton swabs during this period was recorded. The procedure described above was repeated twice, with the mice resting for 10 min between every two experimental intervals. The data were eventually presented as the average time of the mouse sniffing three types of cotton swabs.

### Open-Field Test

Mice were habituated in the testing room and left undisturbed for 30 min prior to testing. Each mouse was placed in the same position of the box at the beginning of the experiment. Data were collected from each mouse placed in the plexiglass open-field arena (40 cm [length] × 40 cm [width] × 30 cm [high]) for a period of 5 min. The any-maze software and supporting camera system automatically recorded the total distance traveled, the number of lines crossed, time spent in the middle zone, and time spent self-grooming of each subject.

### Three-Chamber Sociability Test

The test was performed in a 60 × 40 cm^2^ white plexiglass box divided into three chambers (20 × 40 cm^2^) by plexiglass dividers. The mice could freely move through a small opening (6 × 6 cm) on each divider for 5 min in the empty box to confirm that each animal had no bias for either of the chambers. A sex- and age-matched adult SPF C57 mice was placed in a small cage in one chamber (strange mice chamber), while a small object was placed in another chamber (novel-object chamber) in advance. The tested mice were placed in the center chamber and allowed to travel between chambers for 5 min, and an overhead camera recorded their movements. The data were eventually presented as the time spent in each of the three chambers.

### Enzyme-Linked Immunosorbent Assay

The levels of TNFα, IL-1β, and IL-6 in the maternal serum and fetal brain of the different treatment groups were measured using commercial enzyme-linked immunosorbent assay kits (RayBiotech, ELM-TNFα, ELM-IL1β, and ELM-IL6, respectively). All procedures were performed strictly in accordance with the manufacturer's instructions. Absorbance was measured at a wavelength of 450 nm, and the optical density values were calculated based on standard curves constructed for each assay and performed in duplicate.

### Western Blotting

The total protein extracted from the pre-frontal cortex was used for Western blotting. The membranes were incubated in primary antibodies, including TLR4 (1:1,000, Abcam, CA), NFκB p65 (1:1,000, Cell Signaling Technology, USA), Phospho-NFκB p65 (1:1,000, Cell Signaling Technology, USA), IKKα (1:1,000, Cell Signaling Technology, USA), IBA-1(1:1,000, Wako, Japan), Arg-1 (1:1,000, Abcam, CA), iNOS (1:1,000, Abcam, CA), C3 (1:1,000, Abcam, CA), CR3A (1:1,000, Abways, China), NAMDA2AR (1:1,000, ZENBIO, China), Syn-1 (1:1,000, Abcam, CA), and β-Actin (1:1,000, Santa Cruz, USA) at 4°C overnight, followed by an incubation with HRP-conjugated secondary antibodies (Santa Cruz, USA) at room temperature for 1 h. Protein bands were detected using a chemiluminescent HRP substrate (Millipore, USA). The images were captured using a Syngene GBox Imaging System (Gene Company, China).

### Immunofluorescence Staining

The brain was completely exfoliated and fixed in 4% paraformaldehyde, and subsequently transplanted into 4% paraformaldehyde containing 30% sucrose for dehydration. After the tissue had sank to the bottom, the sections were frozen. Slices with a thickness of 15 μm were treated with 0.3% Triton x-100 for 20 min, and 5% BSA was used to block the samples at room temperature for 1 h. Primary antibodies, including IBA-1 (1:500, Wako, Japan) and TLR4 (1:300, Proteintech, USA) were incubated at 4°C overnight. The fluorescent secondary antibody was incubated at room temperature in the dark for 1 h and was photographed under a Nikon automatic bioluminescence microscope.

### Golgi-Cox Staining

The brain was carefully extracted and washed in PBS, following by an immersion in a 20 mL Golgi-Cox solution and stored for 14 days in the dark. Fourteen days later, the solution was replaced with Solution 3 in the Hito Golgi-Cox OptimStain PreKit and maintained for 3 days. The brain was cut at the coronal plane (200-μm thick). The sections were placed on microslides and the blotted slides were maintained in a humified chamber overnight. The next day, the sections were stained and dehydrated according to the instructions of the Hito Golgi-Cox OptimStain PreKit.

### Statistical Analysis

The data were expressed as the mean ± SEM. Significant differences were calculated via a two-way analysis of variance with a Bonferroni *post-hoc* test with the use of the GraphPad Prism version 5.0 software package. Where there was a statistically significant interaction, all experimental groups were compared using Bonferroni *post-hoc* test. When there was no significant interaction, a Student-Newman-Keuls test was used to analyze the main effect of LPS or TLR4^−/−^. *P*-values of < 0.05 were determined to be statistically significant.

## Results

### Maternal LPS Treatment Increased the Level of Cytokines in the Maternal Serum and Fetal Brain, but Had No Effect on the TLR4^–/–^ Mice

**Figure 1:**
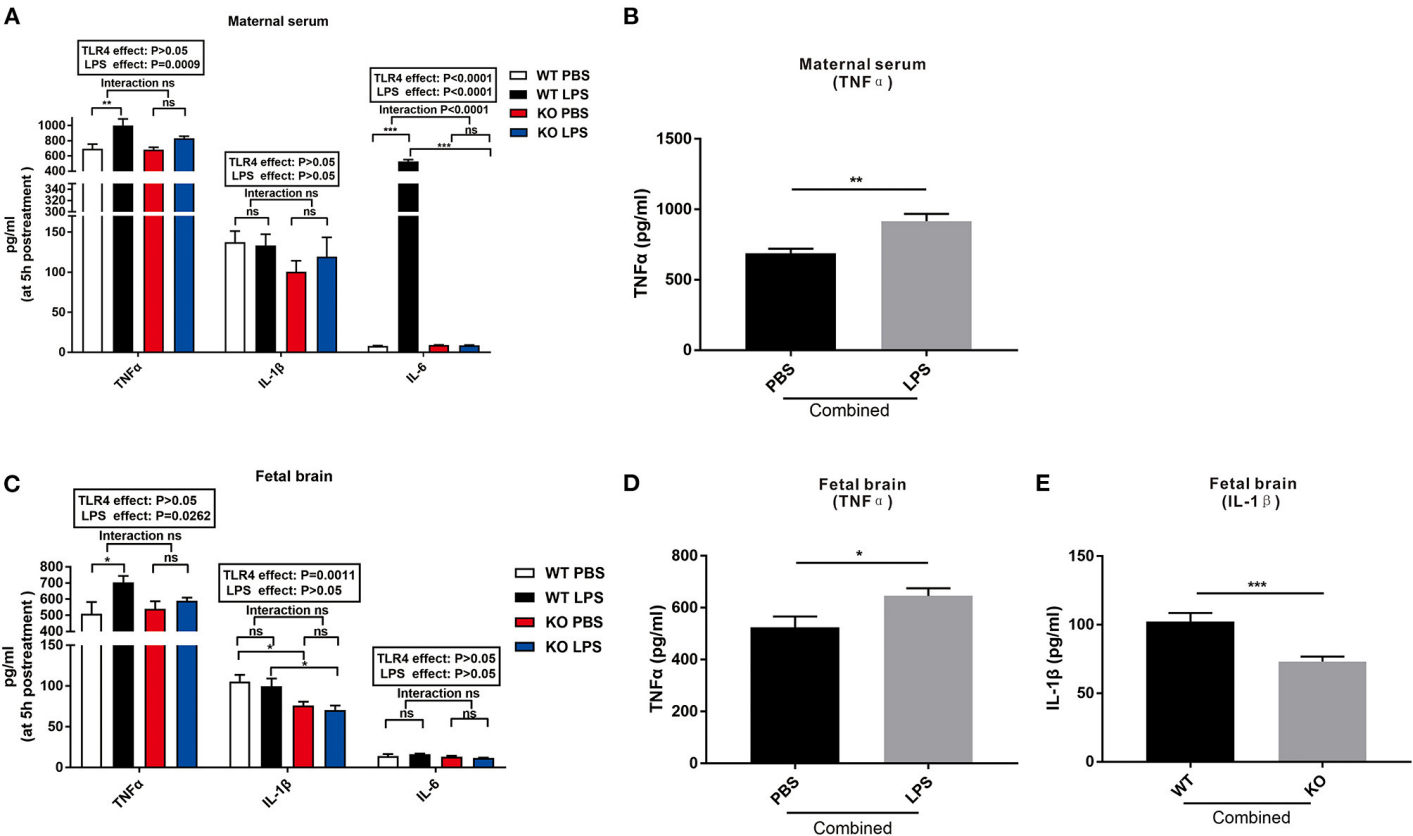
Changes in the levels of TNFα, IL-1β, and IL-6 in the maternal serum and fetal brain at 5 h following LPS treatment in the WT and TLR4^−/−^ mice. **(A)** The effect of PBS and LPS treatment on the levels of TNFα, IL-1β, and IL-6 in the maternal serum in pregnant WT and TLR4^−/−^ mice (*n* = 5). **(B)** The significant effect of LPS, independent of TLR4^−/−^, on the concentration of TNFα in the maternal serum in the combined PBS and LPS groups (*n* = 10). **(C)** Effect of PBS and LPS treatment on the levels of TNFα, IL-1β, and IL-6 in the fetal brain in the WT and TLR4^−/−^ offspring (*n* = 5). **(D)** LPS independently affected the concentrations of TNFα on the fetal brain in the combined PBS and LPS groups (*n* = 10). **(E)** Significant effect of TLR4^−/−^, independent of LPS treatment, on the level of IL-1β on the fetal brain in the combined WT and KO groups (*n* = 10). The values are expressed as the means ± SEMs. “Interaction” indicates an effect of LPS in the TLR4^−/−^ vs. WT mice; ns, not significant, *P < 0.05; **P < 0.01; and ^***^P < 0.001.

To investigate whether LPS treatment during mid-gestation leads to maternal immune activation and determine the fetal response to LPS, the levels of TNFα, IL-1β, and IL-6 in the maternal serum and fetal brain were detected 5 h after LPS exposure. As shown in Figure 1A, LPS exposure and TLR4^−/−^ challenge had no effect on the level of IL-1β in the maternal serum. LPS significantly increased the levels of serum TNFα and IL-6 in the WT pregnant mice (Figure 1A, *P* < 0.01 and *P* < 0.001), but had no effect on the levels of serum TNFα and IL-6 in the TLR4^−/−^ gravid mice (Figure 1A; *P* > 0.05 and *P* > 0.05). After combining the PBS and LPS group, the level of serum TNFα of the maternal mice in the combined LPS group was significantly higher than that in the combined PBS group (Figure 1B; *P* < 0.01).

Figure 1C shows that LPS exposure and TLR4^−/−^ challenge had no effect on the level of IL-6 in the fetal brain. However, LPS exposure for 5 h increased the level of TNFα in the fetal brains of the WT mice compared to that of the TLR4^−/−^ mice (Figure 1C, *P* < 0.05). Further analysis revealed that the level of TNFα in the fetal brain of the combined LPS group was significantly higher than that in the combined PBS group (Figure 1D, *P* < 0.05). Compared with the WT group, the level of IL-1β in the fetal brain of the TLR4^−/−^ group was significantly reduced with or without LPS exposure (Figure 1C, *P* < 0.05). In addition, the level of IL-1β in the fetal brain of the combined WT group was significantly higher than that of the combined TLR4^−/−^ group (Figure 1E, *P* < 0.001). The above data indicate that LPS exposure during pregnancy induced immune activation of WT dams and increased the release of inflammatory factors in the fetal brain, but had no effect on TLR4^−/−^ mice.

### Maternal LPS Treatment Decreased the Litter Size and Weight of Offspring in the WT but Not the TLR4^–/–^ Mice

**Figure 2:**
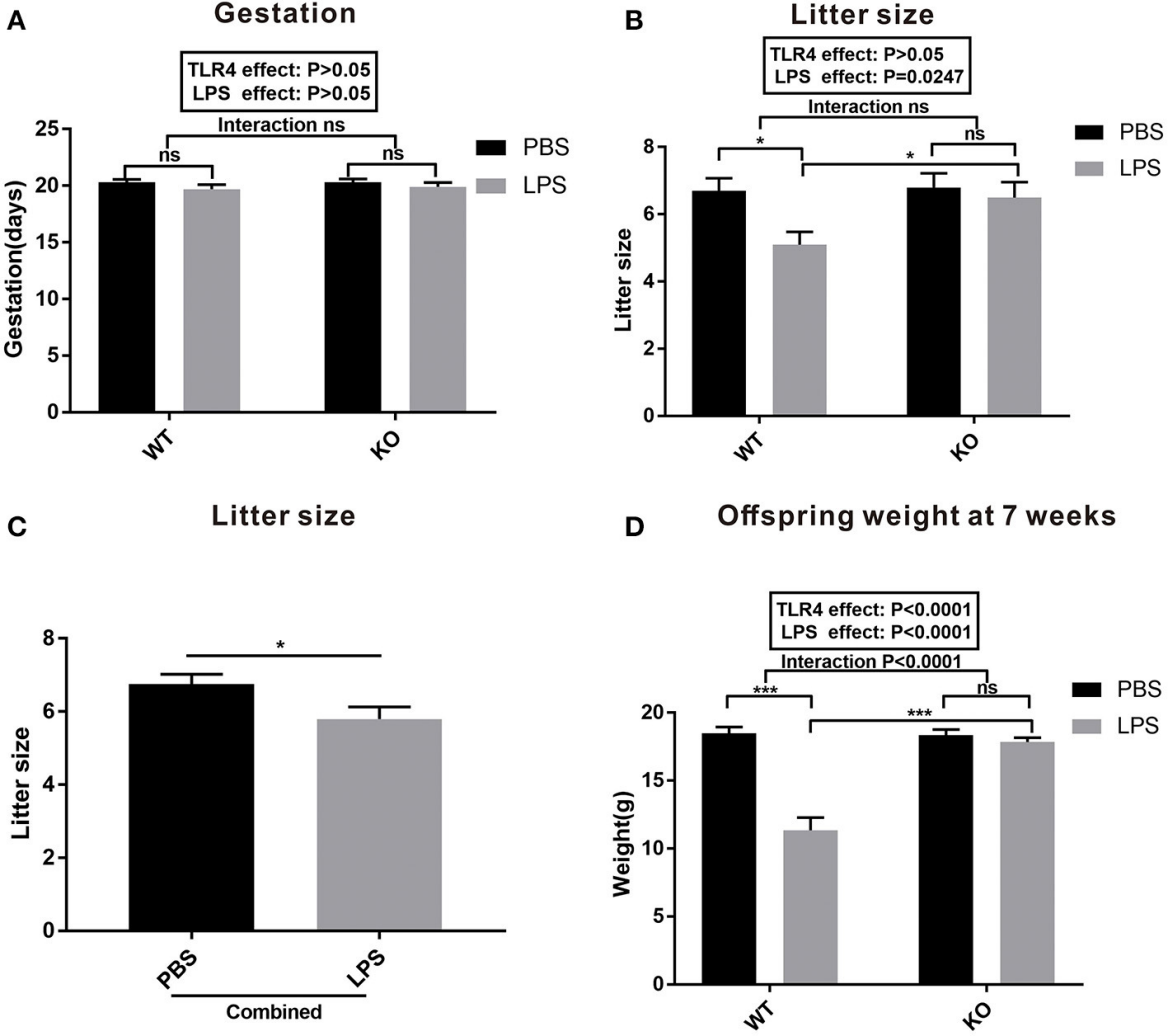
Gestation and litter characteristics. **(A)** Effects of PBS and LPS treatment on the pregnant period between the WT and TLR4^−/−^ pregnant mice (*n* = 10). **(B)** Effects of PBS and LPS treatment on the number of offspring between the WT and TLR4^−/−^ mice (*n* = 10). **(C)** The main effect of LPS, independent of TLR4^−/−^ challenge, on the number of offspring between the combined PBS and LPS groups (*n* = 20). **(D)** Comparison of the effects of PBS and LPS treatment on the weight of offspring between the WT and TLR4^−/−^ mice at 7 weeks of age (*n* = 15). The values are expressed as the means ± SEMs. “Interaction” indicates the effect of LPS in the TLR4^−/−^ vs. WT mice; ns, not significant, *P < 0.05 and ^***^P < 0.001.

To evaluate the effect of LPS exposure during pregnancy on the dams and offspring, we assessed the gestation period, litter size, and offspring weight at seven weeks of age. As shown in Figure 2A, there was no statistical difference in the gestation period between PBS and LPS-exposed dams with or without TLR4^−/−^ challenge (*P* > 0.05). LPS exposure during pregnancy significantly reduced the number of offspring in the WT mice (Figure 2B, *P* < 0.05), but not in TLR4^−/−^ mice (Figure 2B, *P* > 0.05). Further analysis indicated that the litter size in the combined LPS group was significantly lower than that in the combined PBS group (Figure 2C, *P* < 0.05). Figure 2D shows the comparison of the offspring weight at seven weeks of age. The weight in the LPS group was lower than that of the PBS group in the WT mice (*P* < 0.001); however, no significant difference was observed between the PBS and LPS groups in the TLR4^−/−^ mice (*P* > 0.05). A Bonferroni *post-hoc* analysis revealed a significant interaction between the LPS and TLR4^−/−^ challenge (*P* < 0.0001). Therefore, these findings suggest that LPS treatment during pregnancy decreased the number of offspring and weight of the offspring at 7 weeks of age in the WT mice, but had no effect on TLR4^−/−^ mice.

### Maternal LPS Treatment Results in ASD-Like Behavior in the WT Offspring, but Had No Effect on the TLR4^–/–^ Offspring

**Figure 3:**
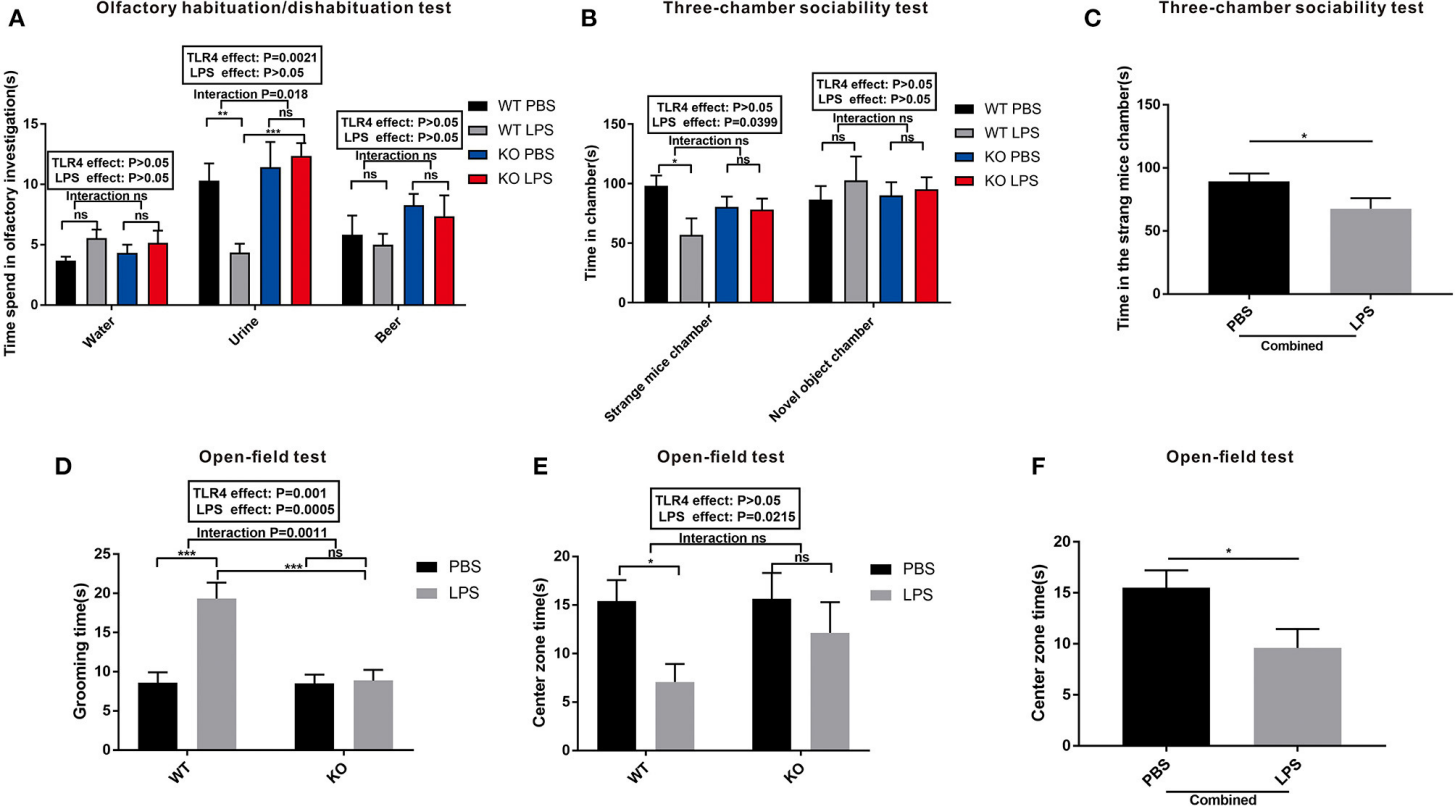
The autism-like behavior tests of the WT and TLR4^−/−^ offspring treated with or without LPS during the gestational period. **(A)** Olfactory habituation/dishabituation test: the cumulative time taken by the offspring mice to sniff cotton swabs. **(B)** Three-chamber sociability test: time spent by the offspring mice in different boxes. **(C)** Significant LPS effect, independent of TLR4^−/−^ challenge, on the amount of time in the stranger mouse chamber between the combined PBS and LPS groups. **(D)** Open-field test: comparison of the self-grooming time of the offspring mice. **(E)** Open-field test: comparison of the time spent in the center zone among the four groups. **(F)** The main effect of LPS, independent of TLR4^−/−^ challenge, on the time spent in the center zone between the combined PBS and LPS groups (*n* = 15). The values are expressed as the means ± SEMs. “Interaction” indicates an effect of the LPS in the TLR4^−/−^ vs. WT mice; ns, not significant, *P < 0.05; **P < 0.01; and ^***^P < 0.001.

The results of the olfactory habituation/dishabituation experiments are shown in Figure 3A. The time spent on the three swabs of distilled water, mouse urine, and beer was compared among the four groups. LPS exposure and TLR4^−/−^ challenge had no effect on the preference of the mice for beer and water. However, the time spent on the swabs of urine in the LPS group was lower than that of the PBS group in the WT offspring (Figure 3A, *P* < 0.01). No significant difference was observed regarding the time spent on the swabs of urine between the KO PBS and KO LPS groups (Figure 3A, *P* > 0.05). These results suggest that LPS treatment during pregnancy did not affect the olfactory ability of WT and TLR4^−/−^ offspring; however, LPS exposure may reduce the interest of WT offspring for their peers, and it had no effect on the TLR4^−/−^ offspring.

A three-chamber sociability test was used to detect the social interaction ability of the mice. As shown in Figure 3B, LPS exposure and TLR4^−/−^ challenge did not affect the duration of the mice in the novel object chamber. However, the LPS-treated offspring showed a much lower preference for the strange mouse chamber compared to PBS-treated offspring in the WT mice (*P* < 0.05). There was no significant difference in the time spent in the strange mouse chamber between the LPS and PBS groups in the TLR4^−/−^ challenge (*P* > 0.05). Although no significant interaction was observed between the LPS and TLR4^−/−^ challenges, the time spent in the strange mouse chamber in the combined LPS group was significantly lower than that of the combined PBS group (Figure 3C, *P* < 0.05). Based on these data, maternal LPS exposure may lead to a social impairment in the WT offspring rather than in the TLR4^−/−^ offspring.

An open-field test is a classical method used to detect the autonomic inquiry activities and the accompanying tension and anxiety of the mice in an unfamiliar environment. Figure 3D shows that the grooming time in the LPS group was greater than that of PBS in the absence of TLR4^−/−^ challenge (*P* < 0.001); however, no significant difference was observed between the PBS and LPS groups with the TLR4^−/−^ challenge (*P* > 0.05). Furthermore, there was no significant interaction between the effects of LPS and TLR4^−/−^ on the time spent in the center zone (Figure 3E). In the absence of TLR4^−/−^, the mice spent less time in the center zone in the LPS group compared with that of the PBS group (Figure 3E, *P* < 0.05). However, there was no statistically significant difference of the mice in the open field center zone between the KO PBS and KO LPS groups (Figure 3E, *P* > 0.05). After combining the PBS and LPS groups, the center zone duration in the combined LPS group was markedly shorter compared with that of the combined PBS group (Figure 3F, *P* < 0.05). These findings suggest that LPS-treated WT offspring had decreased self-exploration behavior, and they exhibited more anxiety and repetitive behaviors in the unfamiliar environment. However, maternal LPS exposure had no effect on the TLR4^−/−^ offspring.

### Maternal LPS Treatment Activated TLR4 and the Microglia Cells of the WT Offspring in Both the Adult and Early Postnatal Period, but Had No Effect on the TLR4^–/–^ Offspring

**Figure 4:**
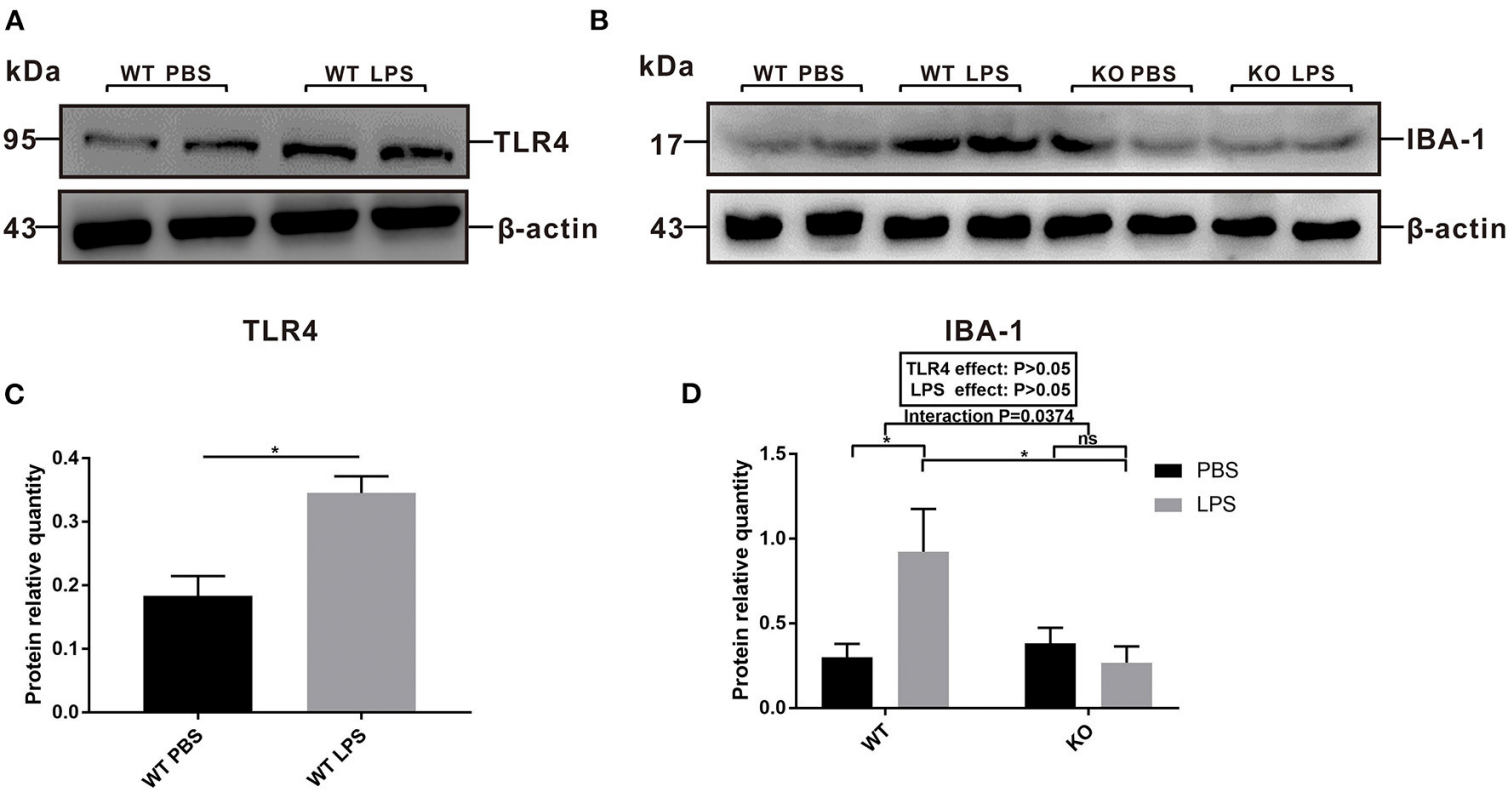
The levels of TLR4 and IBA-1 protein expression in the pre-frontal cortex of 7-week-old WT and TLR4^−/−^ offspring mice treated by PBS or LPS during gestation. **(A)** The level of TLR4 protein expression between the WT PBS and WT LPS groups. **(B)** The level of IBA-1 protein expression in the WT and TLR4^−/−^ mice with or without LPS challenge during pregnancy. **(C)** Quantification analysis of the level of TLR4 protein expression with β-actin normalization. **(D)** Quantification analysis of level of IBA-1 protein expression with β-actin normalization (*n* = 3). The values are expressed as the means ± SEMs. “Interaction” indicates an effect of the LPS in the TLR4^−/−^ vs. WT mice; ns, not significant, *P < 0.05.

To explore the role of TLR4 in LPS-induced maternal immune activation and determine the potential reason for the offspring's ASD-like behavior during adulthood, we examined the levels of TLR4 and IBA-1 protein expression in the pre-frontal cortex of offspring at 7 weeks of age. The results showed that the level of TLR4 expression in the WT LPS group was significantly higher than that of the WT PBS group (Figures 4A,C, *P* < 0.05). In the absence of TLR4^−/−^, maternal LPS exposure significantly increased IBA-1 expression in the pre-frontal cortex of the offspring (Figures 4B,D, *P* < 0.05), and there was no significant difference between the PBS and LPS group with the TLR4^−/−^ challenge (Figures 4B,D, *P* > 0.05). Therefore, LPS treatment during pregnancy led to activation of TLR4 and the microglia cells in the pre-frontal cortex of WT adult offspring; however, microglia were not activated in the TLR4^−/−^ offspring.

**Figure 5:**
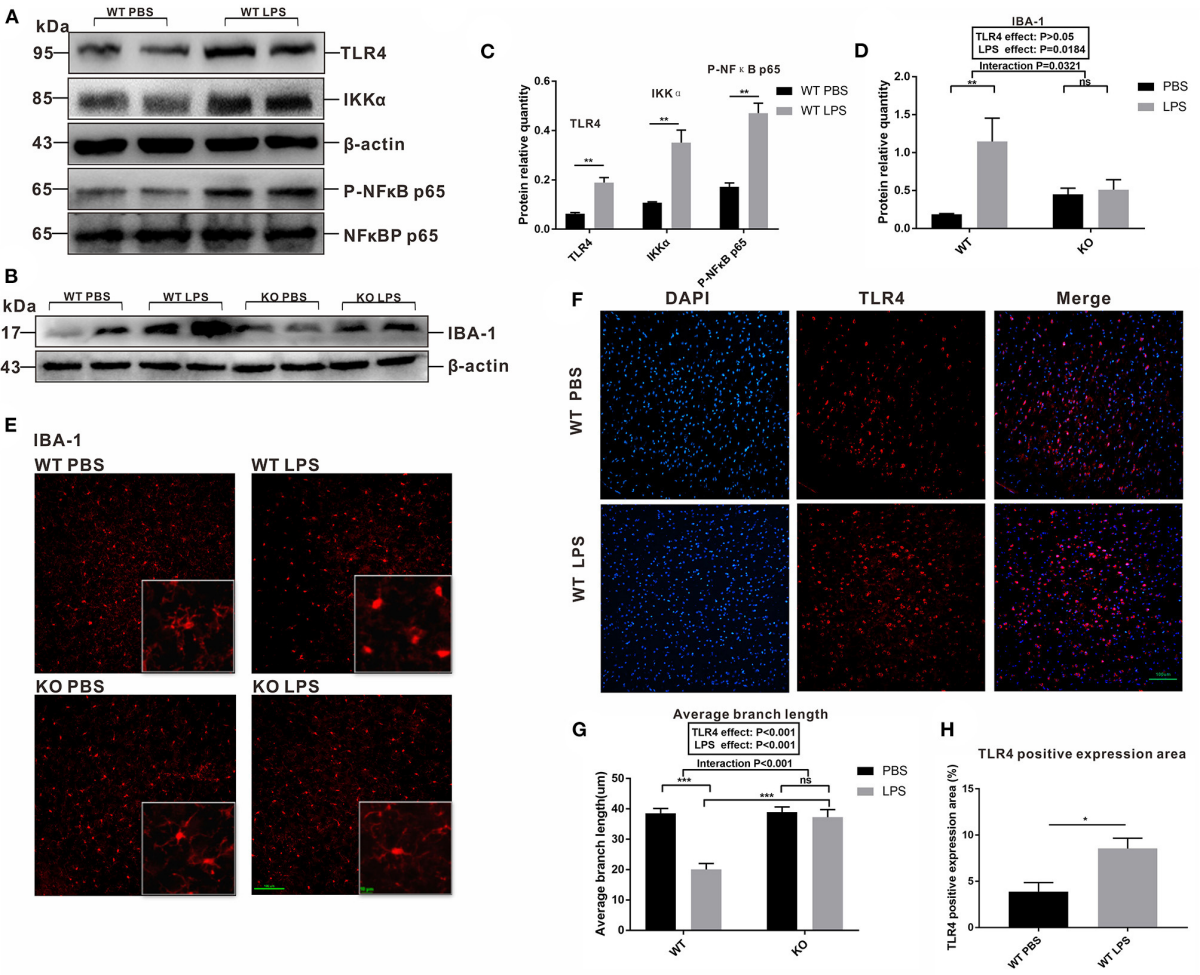
Comparison of the levels of IBA-1, TLR4 and its signaling pathway-associated protein expression, as well as fluctuations in the morphological changes of the microglia in the pre-frontal cortex of 2-week-old offspring mice treated by PBS or LPS during the gestation period. **(A)** The levels of TLR4, Phospho-NFκB p65, and IKKα protein expression between the WT PBS and WT LPS mice. **(B)** The level of IBA-1 protein expression in the PBS and LPS groups with or without TLR4^−/−^ challenge. The quantification analysis of the level of **(C)** TLR4, Phospho-NFκB p65, IKKα, and **(D)** IBA-1 protein expression (*n* = 3). Immunofluorescence staining of **(E)** IBA-1 in the microglia and **(F)** TLR4 expression in different groups (×200). **(G)** The average branch length of the microglia in the PBS and LPS groups with or without TLR4^−/−^ challenge (*n* = 12). **(H)** Quantification analysis of the level of TLR4 expression in immunofluorescence staining (*n* = 3). The values are expressed as the means ± SEMs. “Interaction” indicates an effect of the LPS in the TLR4^−/−^ vs. WT mice; ns, not significant, *P < 0.05; **P < 0.01; and ^***^P < 0.001.

The involvement of microglia in synaptic pruning during early life is crucial for neural development. The peak of microglial density and the optimal time point to detect alterations in synaptic pruning occurred at 15 days after the mice were born (Fernández de Cossío et al., 2017). To investigate whether maternal LPS exposure affects the activation of microglia during the early postnatal period through TLR4, we detected TLR4 signaling pathway-associated proteins and IBA-1 in the pre-frontal cortex of the 2-week old offspring. As shown in the Figures 5A,C, the levels of TLR4, IKKα and Phospho-NFκB p65 protein expression were all increased in the LPS group compared with that of the PBS group in the WT offspring (*P* < 0.01, *P* < 0.01, and *P* < 0.01). Furthermore, the level of IBA-1 protein expression in the LPS group was higher than that of the PBS group in the WT mice (Figures 5B,D, *P* < 0.01). Moreover, maternal LPS treatment had no significant effect on the IBA-1 between the PBS and LPS groups of TLR4^−/−^ mice (Figure 5D, *P* > 0.05), and there was a significant interaction between the LPS and TLR4^−/−^ challenges for IBA-1, according to the *post-hoc* test (*P* = 0.0321).

Our study also observed the morphological changes in the microglia, as well as the expression and distribution of TLR4 in the pre-frontal cortex of the offspring at 2 weeks of age by immunofluorescence staining. As shown in Figure 5E, in the absence of TLR4^−/−^, the microglia body of the PBS group was condensed and obviously multibranched, while the branches of microglia in the LPS group were significantly reduced and shortened. However, the microglia in TLR4^−/−^ mice displayed distinct branches in the pre-frontal cortex with or without LPS treatment. We quantified the average branch length of the microglia cells among the four groups. The average branch length in the LPS group was lower than that of the PBS group in the absence of TLR4^−/−^ (Figure 5G, *P* < 0.001); however, no significant difference was observed between the PBS and LPS groups with the TLR4^−/−^ challenge (Figure 5G, *P* > 0.05), and the average branch length of the KO LPS group was higher than that of the WT LPS group (Figure 5G, *P* < 0.001). Figure 5F compared the fluorescence expression area of TLR4 between the WT PBS group and WT LPS group. The quantified results showed that maternal LPS exposure significantly increased TLR4 expression in the pre-frontal cortex of the offspring (Figure 5H, *P* < 0.05). These results suggest that maternal LPS treatment resulted in microglia activation of the early postnatal period through the TLR4 signaling pathway in the pre-frontal cortex.

### Maternal LPS Treatment Affected Microglial Polarization and Synaptic Pruning-Related Proteins in Early Postnatal WT Mice but Not in the TLR4^–/–^ Offspring

**Figure 6:**
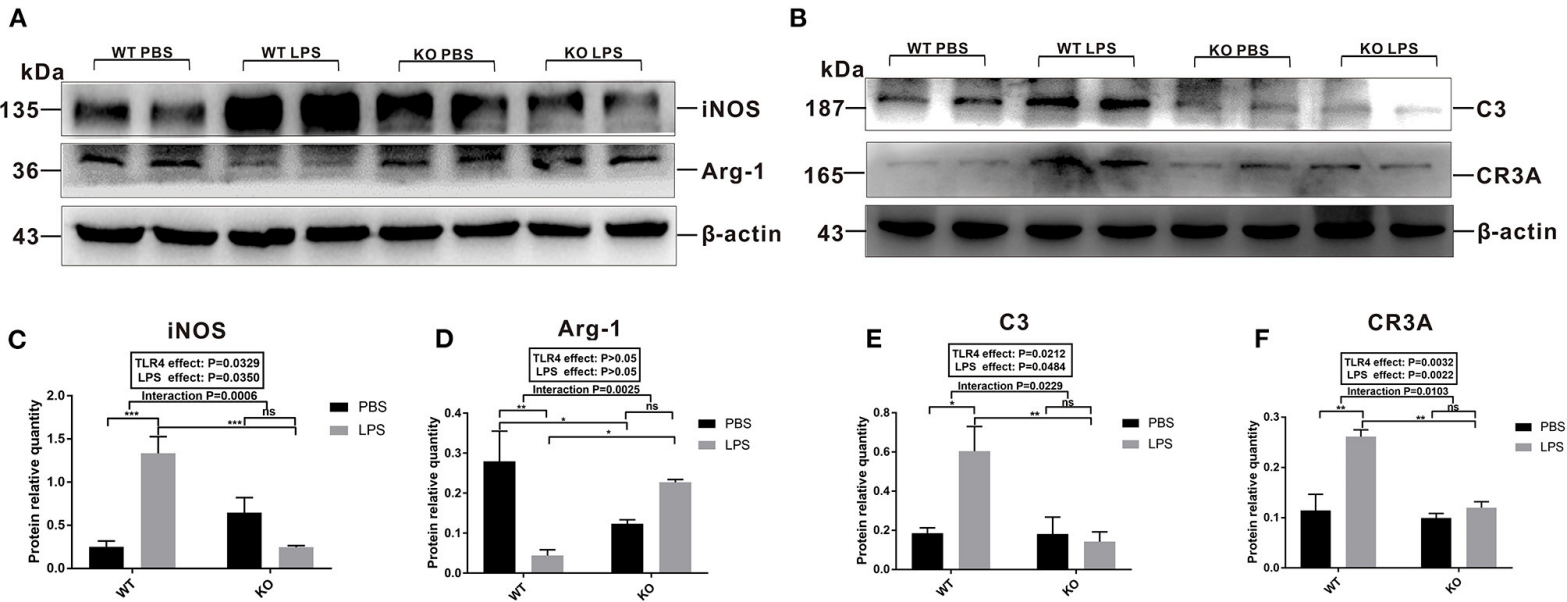
The levels of microglial M1 and M2 polarization and complement C3 pathway-related protein expression in the pre-frontal cortex of 2-week old offspring mice treated with PBS or LPS during gestation. The levels of **(A)** iNOS and Arg-1 **(B)** C3 and CR3A protein expression by Western blot. The quantification analysis of the levels of **(C)** iNOS, **(D)** Arg-1, **(E)** C3, and **(F)** CR3A protein expression with β-actin normalization (*n* = 3). The values are presented as the means ± SEMs. “Interaction” indicates an effect of the LPS in the TLR4^−/−^ vs. WT mice; ns, not significant, *P < 0.05; **P < 0.01; and ^***^P < 0.001.

To explore the specific changes in the M1 and M2 phenotypes of microglia in the offspring's pre-frontal cortex after maternal LPS treatment, we detected the levels of iNOS and Arg-1 expression. The level of iNOS expression in the LPS group was significantly upregulated compared with the PBS group in the absence of TLR4^−/−^ challenge (Figures 6A,C, *P* < 0.001); however, LPS exposure had no significant effect on the level of iNOS expression between the PBS and LPS groups for the TLR4^−/−^ challenge (Figures 6A,C, *P* > 0.05), while TLR4 and LPS had a significant interaction on iNOS expression (*P* = 0.0006). The level of Arg-1 protein expression was significantly down-regulated in the pre-frontal cortex of the WT offspring mice after LPS treatment (Figures 6A,D, *P* < 0.01), whereas there was no significant difference in the pre-frontal cortex of the TLR4^−/−^ offspring mice between the PBS and LPS groups (Figures 6A,D, *P* > 0.05). The TLR4 and LPS interaction had a significant effect on Arg-1 expression (*P* = 0.0025).

We also examined the levels of C3 and CR3A protein expression involved in microglia synapse pruning. As shown in Figures 6B,E, in the absence of TLR4^−/−^, the level of C3 protein expression in the LPS group was higher than that of the mice in the PBS group (Figures 6B,E, *P* < 0.05), and there was no significant difference between the KO PBS and KO LPS groups (Figures 6B,E, *P* > 0.05). The level of CR3A protein expression in the LPS group was higher than that of the PBS group in the WT offspring (Figures 6B,F, *P* < 0.01); however, no significant difference was observed between the PBS and LPS groups with the TLR4^−/−^ challenge (Figures 6B,F, *P* > 0.05). Together, the above results suggest that maternal LPS exposure may lead to M1 subtype polarization of the microglia and enhance the synaptic pruning of the WT offspring at 2 weeks of age, whereas there was no significant effect on the TLR4^−/−^ offspring.

### Effects of Maternal LPS Treatment on the Neuronal Morphology and Synaptic Plasticity-Related Protein Expression During the Early Postnatal Period of the Offspring

**Figure 7:**
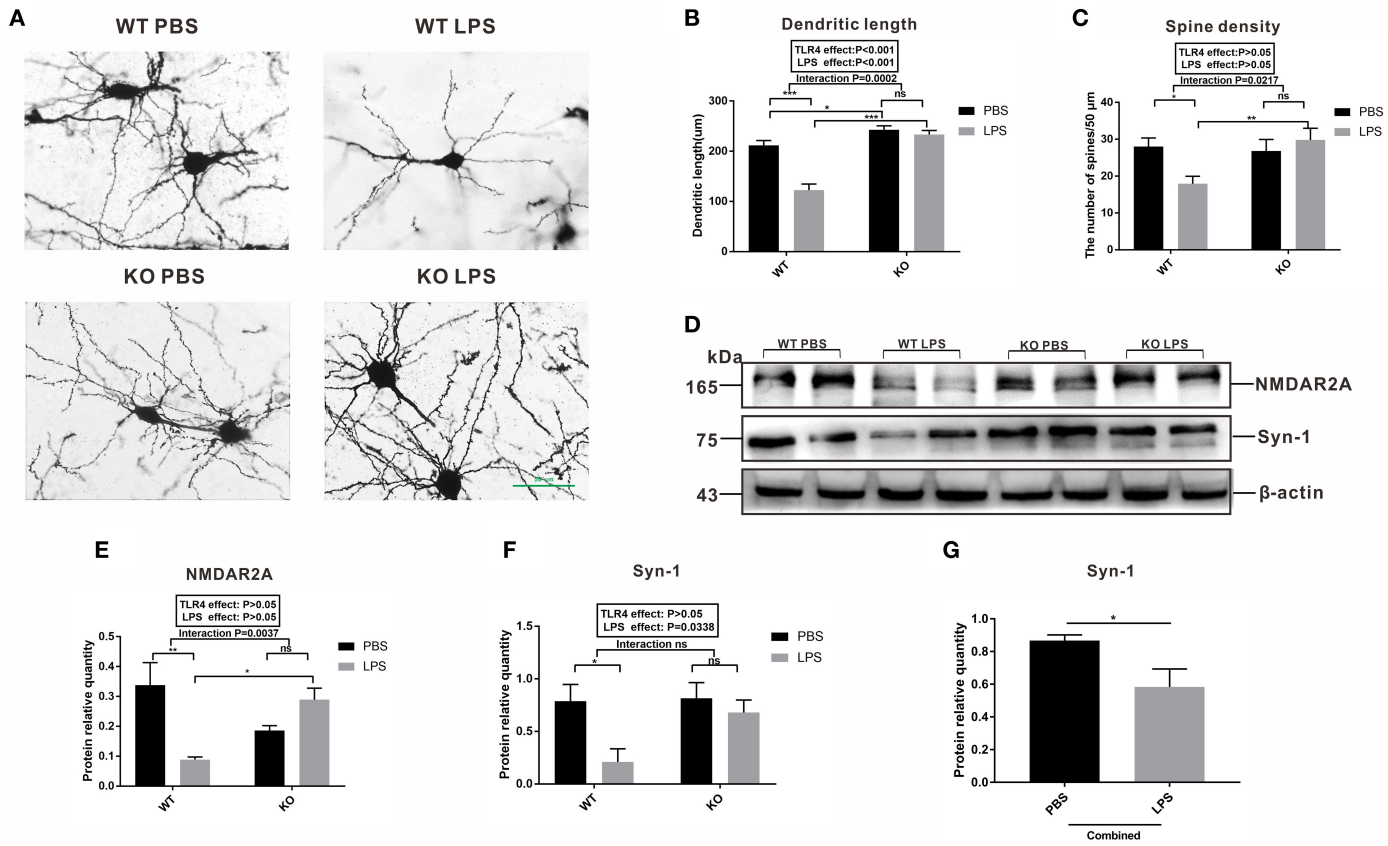
Observation of the neuron synapses and expression of synaptic plasticity-related proteins in the pre-frontal cortex of 2-week old offspring mice treated with PBS or LPS during gestation. **(A)** Photomicrograph showing representative Golgi-Cox impregnation among the four groups (×400). The **(B)** dendritic length and **(C)** the number of spines /50 μm in the PBS and LPS groups with or without TLR4^−/−^ challenge (*n* = 10). **(D)** The levels of NMDAR2A and Syn-1 protein expression in the PBS and LPS groups with or without TLR4^−/−^ challenge. The quantification analysis of the levels of **(E)** NMDAR2A and **(F)** Syn-1 protein expression with β-actin normalization (*n* = 3). **(G)** Significant LPS effect, independent of TLR4^−/−^ challenge, on the level of Syn-1 expression between the combined PBS and LPS groups (*n* = 3). The values are presented as the means ± SEMs. “Interaction” indicates an effect of the LPS in the TLR4^−/−^ vs. WT mice; ns, not significant, *P < 0.05; **P < 0.01; and ***P < 0.001.

To further explore the relationship between microglia activation and the ASD-like behavior induced by maternal LPS exposure, a morphological analysis of the neurons was performed and synaptic plasticity in pre-frontal cortex of 2-week-old offspring mice was examined. Golgi staining was performed on neurons in the pre-frontal cortex of the 2-week old offspring. As shown in Figure 7A, the dendritic branches of the neurons in the WT LPS group were significantly shorter among the four groups. The results of the quantitative analysis showed that the dendrite length of the LPS group was significantly lower than that of the PBS group in the absence of a TLR4^−/−^ challenge (Figure 7B, *P* < 0.001). Moreover, there was no difference in the dendrite length between the PBS group and LPS group with the TLR4^−/−^ challenge (Figure 7B, *P* > 0.05). Furthermore, the dendrite length of the TLR4^−/−^ mice was longer than that of the WT mice with or without LPS treatment (Figure 7B, *P* < 0.05 and *P* < 0.001). At the same time, we also performed a quantitative analysis of the spine density of the neurons. The results showed that in the absence of TLR4^−/−^, the spine density of the LPS group was significantly lower than that of the PBS group (Figure 7C, *P* < 0.05), and there was no statistical difference in the spine density between the LPS group and the PBS group following TLR4^−/−^ challenge (Figure 7C, *P* > 0.05).

Both NMDAR2A and Syn-1 are proteins that represent synaptic plasticity. As shown in Figure 7D, the level of NMDAR2A protein expression in the LPS group was significantly lower than that of the PBS group in the WT mice (Figures 7D,E, *P* < 0.01). There was no statistical difference in the level of NMDAR2A protein expression between the LPS and PBS groups following TLR4^−/−^ challenge (Figures 7D,E, *P* > 0.05). The level of Syn-1 expression in the LPS group was significantly lower than that in the PBS group without TLR4^−/−^ challenge (Figures 7D,F, *P* < 0.05), and there was no statistical difference in the level of Syn-1 protein expression between the KO PBS and KO LPS groups (Figures 7D,F, *P* > 0.05). Further analysis revealed that the level of Syn-1 protein expression was significantly lower in the combined LPS treatment group (Figure 7G, *P* < 0.05) compared to that in the combined PBS group. The above results suggest that maternal LPS exposure impaired the neuronal connections and synaptic plasticity in the WT offspring during the early postnatal period. These results affect the behavior of the mice, whereas maternal LPS exposure had no effect on the TLR4^−/−^ offspring.

## Discussion

Activation of the immune system during pregnancy, combined with genetic and environmental factors, may increase the risk of certain neurodevelopmental disorders in future offspring, including ASD (Meyer, 2019). Animal studies have shown that an excessive maternal immune response during pregnancy is closely related to brain changes and the behavioral development of offspring (Wischhof et al., 2015). LPS, a component of gram-negative bacteria, can induce immune activation and LPS stimulation during pregnancy, representing a classic and mature method of inducing an ASD-like behavior in an animal model (Oskvig et al., 2012; Belle et al., 2014). We observed that maternal LPS exposure could significantly reduce the WT offspring's interest in communication and play with their peers, whereas the stereotypical behavior increased. These findings suggest that the offspring exhibited the same phenotype as children with ASD. Interestingly, LPS had no effect on the behavior of the TLR4^−/−^ offspring. Meyer's study found that pregnant mice exposed to immune stimulation had significantly increased levels of inflammatory cytokines in both the serum and fetal brains (Meyer et al., 2006). Moreover, high circulating levels of the cytokines, tumor necrosis factor-α (TNF-α), interleukin-1β (IL-1β), and IL-6 (Smith et al., 2007; Crampton et al., 2012; Li et al., 2017) in the amniotic fluid and serum are associated with ASD (Olsen et al., 1989; Yoon et al., 1995). Our results are similar to that of Meyer et al.'s (2006), as the levels of serum TNF-α and IL-6 in the WT maternal mice were significantly increased 5 h after LPS exposure. In addition, exposure to LPS during pregnancy significantly increased the level of TNF-α in the fetal brains of WT mice; however, LPS treatment did not increase the levels of TNF-α, IL-1β, and IL-6 in TLR4^−/−^ mice in the maternal serum or fetal brain. The prenatal status and weight of the offspring can reflect early neurological signs(Lucas et al., 1999). Our study found that the duration of pregnancy was not affected by LPS, and the weight of offspring was significantly reduced in the LPS-treated WT offspring, which is consistent with the findings of the study by Fernández de Cossío et al. (2017); however, unlike the Fernandez's study, our research showed that LPS also reduced the number of offspring in WT mice, which may be related to the dose and serotype of LPS. Furthermore, our study demonstrated that LPS had no effect on the number or weight of the TLR4^−/−^ offspring. These data indicate that LPS treatment during pregnancy induced maternal immune activation and increased the fetal brain inflammatory response in WT mice. In addition, the number and the weight of offspring in the WT mice were significantly decreased. Following LPS stimulation during pregnancy, the offspring of WT mice showed significant ASD characteristics, suggesting that an animal model of LPS-induced offspring with ASD-like behavior was successfully constructed. In contrast, TLR4^−/−^ mice lack an inflammatory response in maternal serum and fetal brain, and the TLR4^−/−^ offspring did not exhibit ASD-like behavior following maternal LPS stimulation, indicating that TLR4 is a key contributor to ASD-like behavior in offspring due to maternal LPS exposure.

TLRs are a key family of membrane-anchored proteins expressed on immune cells and enterocytes (Xiao et al., 2019). Among these, TLR4 recognizes pathogen-related molecular patterns, including the specific recognition of LPS, and stimulates a series of complex pro-inflammatory cascade reactions by activating the TLR4/NFκB signaling pathway, leading to disease progression (Fernandez-lizarbe et al., 2009; Lucas and Maes, 2013; García-bueno et al., 2016). It has been reported that circulating LPS may lead to abnormal behavior by activating TLR4 in the CNS, whereas blocking LPS-induced TLR4 activation will suppress the transmission of brain immune signals and prevent disease occurrence (Hines et al., 2013). Our results showed that the TLR4 signaling pathway was activated in the pre-frontal cortex of the WT offspring after maternal LPS stimulation, whether it was a 7-week-old adult offspring or a 2-week-old offspring. This effect was not induced in the TLR4^−/−^ offspring, indicating that TLR4 may represent a link between maternal immune activation and ASD-like behavior in mice.

As the main immune cell in the CNS, microglia represent the first line of defense for immune activation and injury (Ginhoux et al., 2010; Pascual et al., 2012). In the CNS, TLR4 is primarily expressed in microglia cells, involved in microglia-mediated inflammatory responses, and leads to cytokine production and secretion (Bueno et al., 2016). The study by Fernandez-lizarbe et al. (2009) showed that TLR4 plays a crucial role in the animal models of neuroinflammatory damage caused by alcohol-induced microglial activation. Activated microglia have two phenotypes, M1 and M2. The M1 phenotype induces the secretion of pro-inflammatory cytokines and inflammatory mediators (e.g., IL-6, TNFα, and iNOS), causing tissue damage, whereas the M2 phenotype secretes anti-inflammatory cytokines and Arg-1 to reduce the acute inflammatory response (Microglial et al., 2016). A large number of studies support the activation and increased number of microglia in the pre-frontal cortex of ASD patients (Morgan et al., 2010; Edmonson et al., 2014). In LPS-induced ASD-like behavior animal models, microglia activation has been demonstrated by numerous studies, exhibited by the upregulation of IBA-1, increased release of iNOS, and morphological changes (Juckel et al., 2011; Cunningham et al., 2013). Our results are consistent with the above studies, as the microglia polarized toward M1 phenotype following maternal LPS stimulation in WT mice. Interestingly, LPS could not activate the microglia in the pre-frontal cortex of the TLR4^−/−^ offspring, which suggests that LPS treatment during pregnancy induces an ASD-like behavior in adult offspring that requires the TLR4 signaling pathway to activate the microglia in the pre-frontal cortex.

Recent studies have revealed that abnormally activated microglia can interfere with neural circuits through the phagocytosis and clearance of synaptic structures (dendritic spines), affecting synaptic plasticity, leading to neurodegenerative disease (Bilimoria and Stevens, 2015). During normal development, high CR3 expression in microglia cells recognizes C3 expression on immature neurons and prunes the dendrites of neurons to maintain normal connections between neurons, whereas abnormal microglia activation may affect the processing of synaptic pruning, resulting in neurological dysfunction (Matcovitch-Natan et al., 2016). Animal experiments confirm that maternal LPS exposure regulates the expression of genes related to synapse pruning in mice (Fernández de Cossío et al., 2017) and microglia synaptic pruning defects may be involved in the pathogenesis of ASD (Matcovitch-Natan et al., 2016). Despite some findings to the contrary, most studies show that maternal LPS exposure leads to a decrease in the number of dendritic spines and a shorter dendritic length in the offspring brain tissue (Baharnoori et al., 2009). Since the peak of microglia synaptic pruning and neuronal maturation occurred 5–15 days after the mice were born (Matcovitch-Natan et al., 2016; Fernández de Cossío et al., 2017), our study detected synaptic pruning-associated proteins in the pre-frontal cortex of 2-week-old mice. The level of C3 and CR3A expression in the WT mice was significantly increased following maternal LPS stimulation, whereas the dendrite length and spine density were significantly decreased. However, maternal LPS treatment did not affect the level of C3 and CR3A protein expression in the TLR4^−/−^ offspring, nor did it reduce the length of neuronal dendrites and the density of the dendritic spines. These findings reveal that TLR4 is a key molecule involved in the synaptic pruning associated with LPS-induced microglia activation.

The synaptic proteins, NMDAR2A (N-methyl-D-aspartate receptors 2A) and Syn-1 (Synapsin1), play a key role in regulating neuron survival, which affect the development of dendrites and participate in the formation of synaptic plasticity (Forrest et al., 2012; Montesinos et al., 2015). The study by Forrest et al. found that maternal immune activation reduces the expression of the NMDA receptor subunits in the brains of offspring mice, suggesting that synaptic plasticity is impaired during brain development, which is consistent with the findings of our study (Forrest et al., 2012). Studies have shown that TLR4 impacts synaptic plasticity by regulating the expression of synaptic proteins, and treatment with a TLR4 antagonist can prevent decreased NR2A expression (Montesinos et al., 2015); however, the specific mechanism remains unclear. Our research results show that the levels of NMDAR2A and Syn-1 expression in WT mice were significantly decreased following maternal LPS stimulation, whereas LPS did not affect the level of NMDAR2A and Syn-1 protein expression in the TLR4^−/−^ offspring, suggesting that TLR4 represents a key factor in the changes in synaptic plasticity in the offspring of LPS-induced ASD animal models.

Therefore, these results provide the first evidence of the role of TLR4 in microglia activation, leading to ASD-like behavior of offspring induced by maternal LPS exposure. However, it remains unclear how the TLR4 signaling pathway regulates microglial polarization from our study. So, studying the specific molecular mechanism of TLR4 in microglia activation is the next focus of our team.

## Conclusions

**Figure 8:**
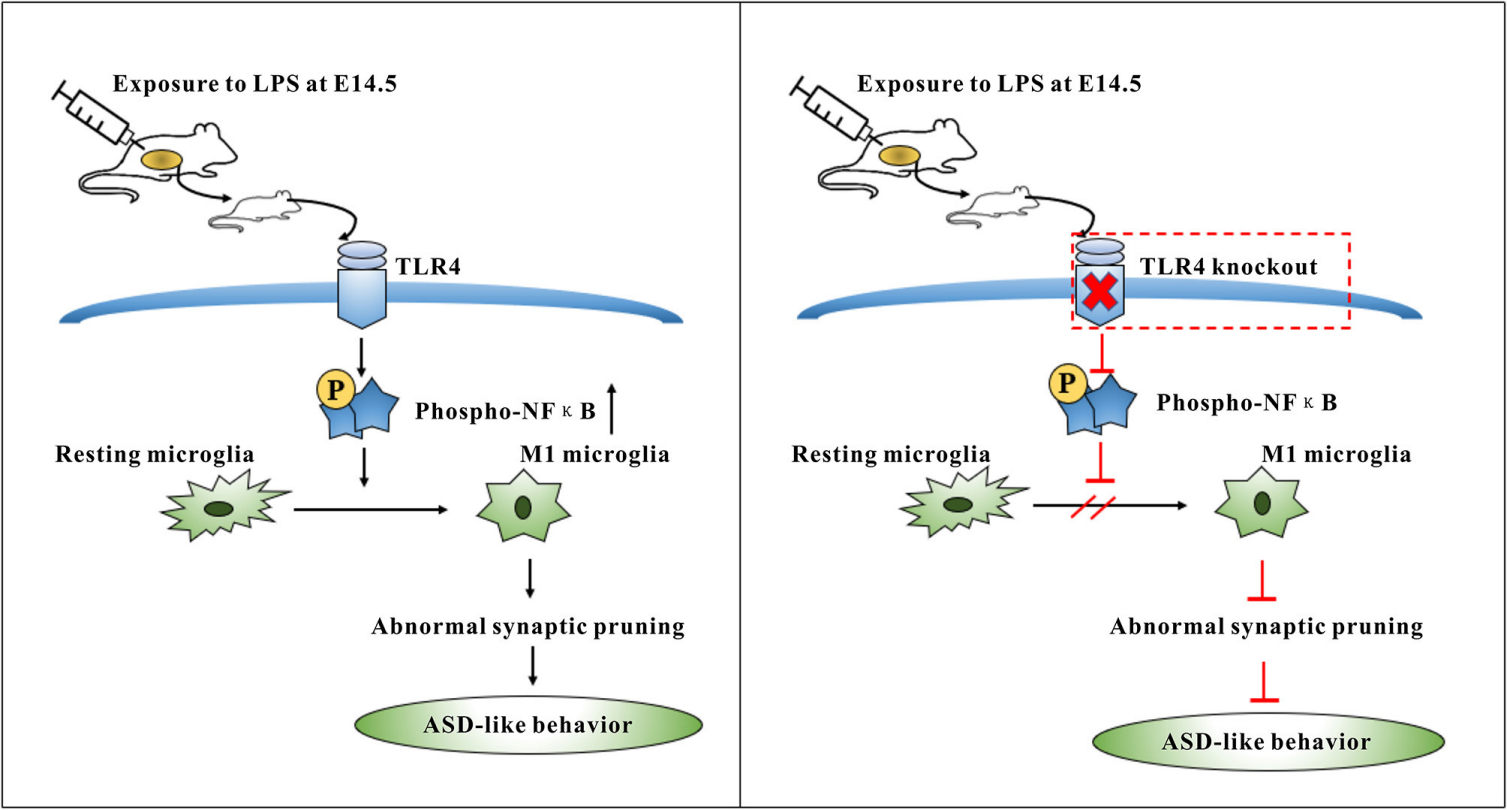
Summary diagram showing the role of TLR4 signaling pathway in the microglia activation induced by maternal LPS exposure leading to ASD-like behavior of offspring. TLR4-deficient mice can protect against ASD-like behavior of offspring by maternal LPS exposure through inhibiting the M1 type polarization and abnormal synaptic pruning of microglia.

This study suggested that activation of the TLR4 signaling pathway in the offspring induced abnormal microglia activation, which could, in turn, involve in excessive synaptic pruning and leading to decreased synaptic plasticity (Figure 8). This may be one of the reasons for ASD-like behavior in offspring mice exposed to maternal LPS. The findings presented herein provide new clues for further study of ASD pathogenesis.

## Data Availability Statement

The raw data supporting the conclusions of this article will be made available by the authors, without undue reservation.

## Ethics Statement

The animal study was reviewed and approved by the Animal Experimentation Ethics Committee of Chongqing Medical University (Chongqing, China) and conducted in accordance with the guidelines of the Animal Care Committee of Chongqing Medical University.

## Author Contributions

LX performed the experiment and analyzed the data. JY, DF, and SY assisted in the completion of the experiment. TY provided technical guidance. LX and JC wrote the manuscript. WS and JC designed the study. JC and HW provided financial support for the study. All authors read and approved the final manuscript.

## Conflict of Interest

The authors declare that the research was conducted in the absence of any commercial or financial relationships that could be construed as a potential conflict of interest.

## References

[B1] Baharnoori M. Brake W. G. Srivastava L. K. (2009). Prenatal immune challenge induces developmental changes in the morphology of pyramidal neurons of the prefrontal cortex and hippocampus in rats. Schizophr. Res. 107, 99–109. 10.1016/j.schres.2008.10.003 19004618

[B2] Belle J. E. Le S. J. Ngo A. Ghochani Y. Laks D. R. Lo M.. (2014). Maternal inflammation contributes to brain overgrowth and autism- associated behaviors through altered redox signaling in stem and progenitor cells. Stem Cell Reports 3, 725–734. 10.1016/j.stemcr.2014.09.004 25418720 PMC4235743

[B3] Bilbo S. D. Block C. L. Bolton J. L. Hanamsagar R. Tran P. K. (2018). Beyond infection - maternal immune activation by environmental factors, microglial development, and relevance for autism spectrum disorders. Exp. Neurol. 299, 241–251. 10.1016/j.expneurol.2017.07.002 28698032 PMC5723548

[B4] Bilimoria P. M. Stevens B. (2015). Microglia function during brain development: new insights from animal models. Brain Res. 1617, 7–17. 10.1016/j.brainres.2014.11.032 25463024

[B5] Bueno B. G. Caso J. R. Madrigal J. L. M. Leza J. C. (2016). Neuroscience and biobehavioral reviews innate immune receptor toll-like receptor 4 signalling in neuropsychiatric diseases. Neurosci. Biobehav. Rev. 64, 134–147. 10.1016/j.neubiorev.2016.02.013 26905767

[B6] Chen Z. Jalabi W. Shpargel K. B. Farabaugh K. T. Dutta R. Yin X.. (2012). Lipopolysaccharide-induced microglial activation and neuroprotection against experimental brain injury is independent of hematogenous TLR4. J. Neurosci. 32, 11706–11715. 10.1523/JNEUROSCI.0730-12.2012 22915113 PMC4461442

[B7] Crampton S. J. Collins L. M. Toulouse A. Nolan Y. M. Gerard W. (2012). Exposure of foetal neural progenitor cells to IL-1 impairs their proliferation and alters their differentiation – a role for maternal inflammation? J. Neurochem. 120, 964–973. 10.1111/j.1471-4159.2011.07634.x 22192001

[B8] Cunningham C. L. Martínez-Cerdeño V. Noctor S. C. (2013). Microglia regulate the number of neural precursor cells in the developing cerebral cortex. J. Neurosci. 33, 4216–4233. 10.1523/JNEUROSCI.3441-12.2013 23467340 PMC3711552

[B9] Edmonson C. Ziats M. N. Rennert O. M. (2014). Altered glial marker expression in autistic post-mortem prefrontal cortex and cerebellum. Mol. Autism 5, 1–9. 10.1186/2040-2392-5-3 24410870 PMC3914711

[B10] Fernández de Cossío L. Guzmán A. van der Veldt S. Luheshi G. N. (2017). Prenatal infection leads to ASD-like behavior and altered synaptic pruning in the mouse offspring. Brain Behav. Immun. 63, 88–98. 10.1016/j.bbi.2016.09.028 27697456

[B11] Fernandez-lizarbe S. Pascual M. Guerri C. (2009). Critical role of TLR4 response in the activation of microglia induced by ethanol. J. Immunol. 183, 4733–4744. 10.4049/jimmunol.0803590 19752239

[B12] Filiano A. J. Gadani S. P. Kipnis J. Scientist M. Program T. (2016). Interactions of innate and adaptive immunity in brain development and function Anthony. Brain Res. 1617, 18–27. 10.1016/j.brainres.2014.07.050 PMC4320678 25110235

[B13] Forrest C. M. Khalil O. S. Pisar M. Smith R. A. Darlington L. G. Stone T. W. (2012). Prenatal activation of Toll-like receptors-3 by administration of the viral mimetic poly(I:C) changes synaptic proteins, N-methyl-D-aspartate receptors and neurogenesis markers in offspring. Mol. Brain 5:22. 10.1186/1756-6606-5-22 22681877 PMC3496691

[B14] Fortier M. E. Luheshi G. N. Boksa P. (2007). Effects of prenatal infection on prepulse inhibition in the rat depend on the nature of the infectious agent and the stage of pregnancy. Behav. Brain Res. 181, 270–277. 10.1016/j.bbr.2007.04.016 17553574

[B15] García-bueno B. Gassó P Macdowell K. S. Callado L. F. Mas S. Bernardo M.. (2016). Evidence of activation of the Toll-like receptor-4 proinflammatory pathway in patients with schizophrenia. J. Psychiatry Neurosci. 41, 46–55. 10.1503/jpn.150195 27070349 PMC4853215

[B16] Ginhoux F. Greter M. Leboeuf M. Nandi S. See P. Gokhan S.. (2010). Fate mapping analysis reveals that adult microglia derive from primitive macrophages. Science 330, 841–845. 10.1126/science.1194637 20966214 PMC3719181

[B17] Hines D. J. Choi H. B. Hines R. M. Phillips A. G. Macvicar B. A. (2013). Prevention of LPS-induced microglia activation, cytokine production and sickness behavior with TLR4 receptor interfering peptides. PLoS ONE 8:e60388. 10.1371/journal.pone.0060388 23555964 PMC3610686

[B18] Hromada-judycka A. (2015). Co-operation of TLR4 and raft proteins in LPS-induced pro-inflammatory signaling. Cell. Mol. Life Sci. 72, 557–581. 10.1007/s00018-014-1762-5 25332099 PMC4293489

[B19] Hyman S. L. Levy S. E. Myers S. M. (2020). Identification, evaluation, and management of children with autism spectrum disorder. Pediatrics 145:e20193447. 10.1542/peds.2019-3447 31843864

[B20] Juckel G. Manitz M. P. Brüne M. Friebe A. Heneka M. T. Wolf R. J. (2011). Microglial activation in a neuroinflammational animal model of schizophrenia - a pilot study. Schizophr. Res. 131, 96–100. 10.1016/j.schres.2011.06.018 21752601

[B21] Lehnardt S. Lachance C. Patrizi S. Lefebvre S. Follett P. L. Jensen F. E.. (2002). The toll-like receptor TLR4 is necessary for lipopolysaccharide- induced oligodendrocyte injury in the CNS. J. Neurosci. 22, 2478–2486. 10.1523/JNEUROSCI.22-07-02478.2002 11923412 PMC6758325

[B22] Li Q. Han Y. Dy A. B. C. Hagerman R. J. (2017). The gut microbiota and autism spectrum disorders. Front. Cell. Neurosci. 11:120. 10.3389/fncel.2017.00120 28503135 PMC5408485

[B23] Lucas A. Fewtrell M. S. Cole T. J. (1999). Fetal origins of adult disease-the hypothesis revisited. BMJ 319, 245–249. 10.1136/bmj.319.7204.245 10417093 PMC1116334

[B24] Lucas K. Maes M. (2013). Role of the Toll Like Receptor (TLR) radical cycle in chronic inflammation : possible treatments targeting the TLR4 pathway. Mol. Neurobiol. 48, 190–204. 10.1007/s12035-013-8425-7 23436141 PMC7091222

[B25] Matcovitch-Natan O. Winter D. R. Giladi A. Aguilar S. V. Spinrad A. Sarrazin S.. (2016). Microglia development follows a stepwise program to regulate brain homeostasis. Science 353:aad8670. 10.1126/science.aad8670 27338705

[B26] Mcdougle C. J. Landino S. M. Vahabzadeh A. Rourke J. O. Zurcher N. R. Finger B. C.. (2015). Toward an immune-mediated subtype of autism spectrum disorder. Brain Res. 1617, 72–92. 10.1016/j.brainres.2014.09.048 25445995

[B27] Meyer U. (2019). Neurodevelopmental resilience and susceptibility to maternal immune activation. Trends Neurosci. 42, 793–806. 10.1016/j.tins.2019.08.001 31493924

[B28] Meyer U. Nyffeler M. Engler A. Urwyler A. Schedlowski M. Knuesel I.. (2006). The time of prenatal immune challenge determines the specificity of inflammation-mediated brain and behavioral pathology. J. Neurosci. 26, 4752–4762. 10.1523/JNEUROSCI.0099-06.2006 16672647 PMC6674174

[B29] Microglial M. Orihuela R. Mcpherson C. A. Harry G. J. (2016). Microglial M1/M2 polarization and metabolic states. Br. J. Pharmacol. 173, 649–665. 10.1111/bph.13139 25800044 PMC4742299

[B30] Montesinos J. Pascual M. Pla A. Maldonado C. Rodríguez-Arias M. Miñarro J.. (2015). TLR4 elimination prevents synaptic and myelin alterations and long-term cognitive dysfunctions in adolescent mice with intermittent ethanol treatment. Brain Behav. Immun. 45, 233–244. 10.1016/j.bbi.2014.11.015 25486089

[B31] Morgan J. T. Chana G. Pardo C. A. Achim C. Semendeferi K. Buckwalter J.. (2010). Microglial activation and increased microglial density observed in the dorsolateral prefrontal cortex in autism. BPS 68, 368–376. 10.1016/j.biopsych.2010.05.024 20674603

[B32] Olsen K. D. Reisch J. S. Beutler B. Mccracken G. H. (1989). Correlation of interleukin- / 3 and cachectin concentrations in cerebrospinal fluid and outcome from bacterial meningitis. J. Pediatr. 115, 208–213. 10.1016/S0022-3476(89)80067-8 2787856

[B33] Oskvig D. B. Elkahloun A. G. Johnson K. R. Phillips T. M. Herkenham M. (2012). Maternal immune activation by LPS selectively alters specific gene expression profiles of interneuron migration and oxidative stress in the fetus without triggering a fetal immune response. Brain Behav. Immun. 26, 623–634. 10.1016/j.bbi.2012.01.015 22310921 PMC3285385

[B34] Pascual O. Achour S. Ben R. P. Triller A. Bessis A. (2012). Microglia activation triggers astrocyte-mediated modulation of excitatory neurotransmission. Proc. Natl. Acad. Sci. U. S. A. 109, 197–205. 10.1073/pnas.1111098109 22167804 PMC3268269

[B35] Ransohoff R. M. Cardona A. E. (2010). The myeloid cells of the central nervous system parenchyma. Nature 468, 253–262. 10.1038/nature09615 21068834

[B36] Salter M. W. Stevens B. (2017). Microglia emerge as central players in brain disease. Nat. Med. 23, 1018–1027. 10.1038/nm.4397 28886007

[B37] Santra A. Timi T. Stankovi T. (2016). Lipopolysaccharide exposure during late embryogenesis results in diminished locomotor activity and amphetamine response in females and spatial cognition impairment in males in adult, but not adolescent rat offspring. Behav. Brain Res. 299, 72–80. 10.1016/j.bbr.2015.11.025 26620494

[B38] Schafer D. P. Lehrman E. K. Stevens B. (2013). The “quad-partite” synapse: microglia-synapse interactions in the developing and mature CNS. Glia 61, 24–36. 10.1002/glia.22389 22829357 PMC4082974

[B39] Smith S. E. P. Li J. Garbett K. Mirnics K. Patterson P. H. (2007). Maternal immune activation alters fetal brain development through interleukin-6. J. Neurosci. 27, 10695–10702. 10.1523/JNEUROSCI.2178-07.2007 17913903 PMC2387067

[B40] Takeda K. Akira S. (2015). Toll-like receptors. Curr. Prot. Immunol. 2015, 14.12.1–14.12.10. 10.1002/0471142735.im1412s109 25845562

[B41] Wischhof L. Irrsack E. Osorio C. Koch M. (2015). Prenatal LPS-exposure - a neurodevelopmental rat model of schizophrenia - differentially affects cognitive functions, myelination and parvalbumin expression in male and female offspring. Prog. Neuro-Psychopharmacol. Biol. Psychiatry 57, 17–30. 10.1016/j.pnpbp.2014.10.004 25455585

[B42] Xiao L. Chen B. Feng D. Yang T. Li T. Chen J. (2019). TLR4 may be involved in the regulation of colonic mucosal microbiota by vitamin A. Front Microbiol. 10:268. 10.3389/fmicb.2019.00268 30873131 PMC6401601

[B43] Yoon B. H. Romero R. Kim C. J. Jun J. K. Gomez R. Choi J. H.. (1995). Amniotic fluid interleukin-6 : a sensitive test for antenatal diagnosis of acute inflammatory lesions of preterm placenta and prediction of perinatal morbidity. Am. J. Obstet. Gynecol. 172, 960–970. 10.1016/0002-9378(95)90028-4 7892891

