# Supplementary material for: Critical Role of TLR4 on the Microglia Activation Induced by Maternal LPS Exposure Leading to ASD-Like Behavior of Offspring
Source: Front Cell Dev Biol. 2021 Mar 4;9:634837. doi: 10.3389/fcell.2021.634837 (PMC7969707; doi:10.3389/fcell.2021.634837)
Supplement: Supplementary file 1 [file Data_Sheet_1.doc]

**Critical role of TLR4 on the microglia activation induced by maternal LPS exposure leading to ASD-like behavior of offspring**

Lu Xiao, Junyan Yan, Di Feng, Shasha Ye, Ting Yang, Hua Wei, Tingyu Li, Wuqing Sun^*^, Jie Chen^*^

**Supplementary material**

**Figure 1**


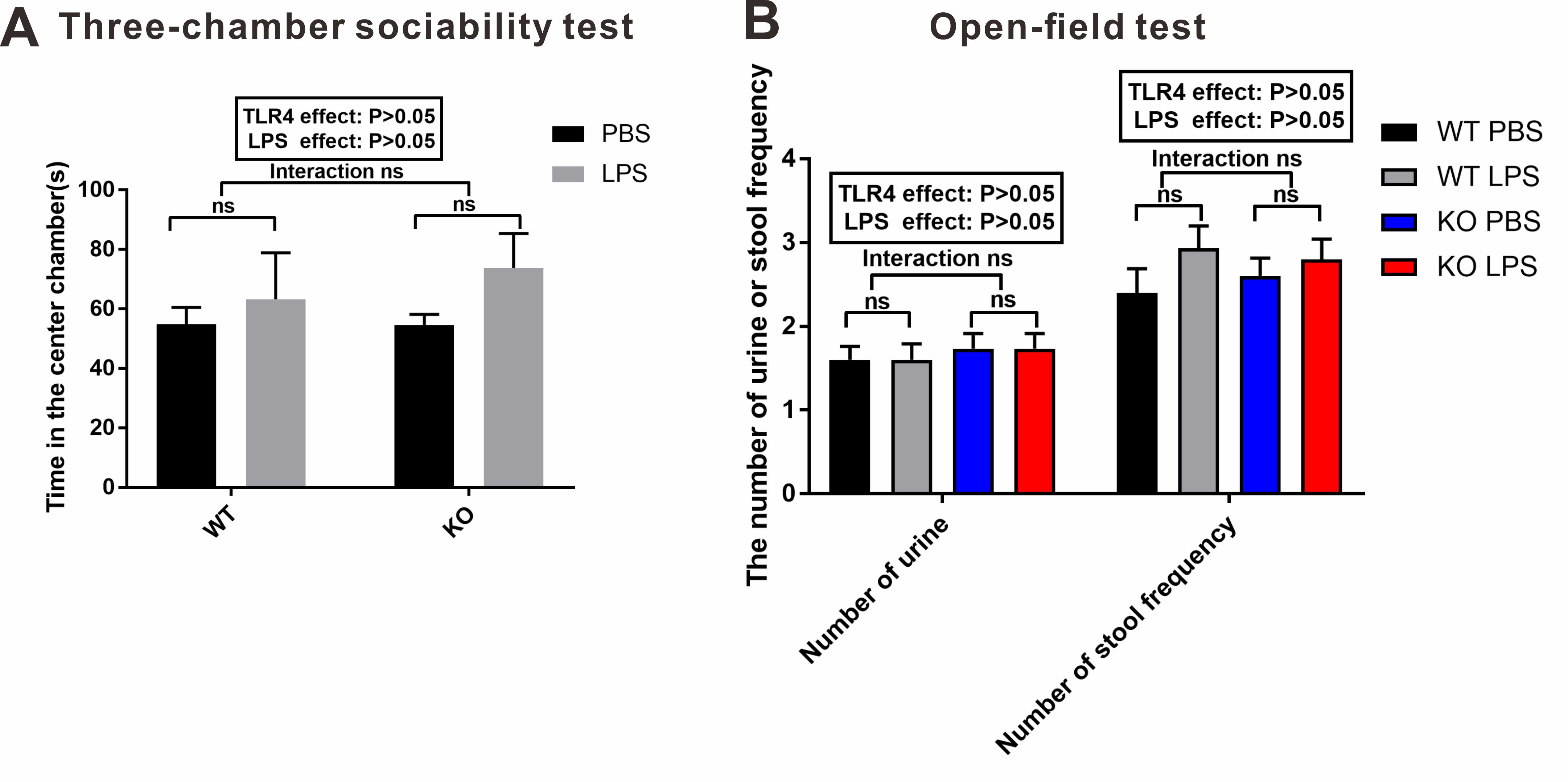


Figure 1. The autism-like behavior tests of the WT and TLR4^-/-^ offspring treated with or without LPS during gestation. (A) Three-chamber sociability test: time spent by the offspring mice in the center chamber. (B) Open-field test: the comparisons of the urine and stool frequency among the four groups (n = 15). The values are expressed as the means ± SEMs. “Interaction” indicates an effect of the LPS in the TLR4^-/-^ vs. WT mice; ns, not significant.

**Figure 2**

**
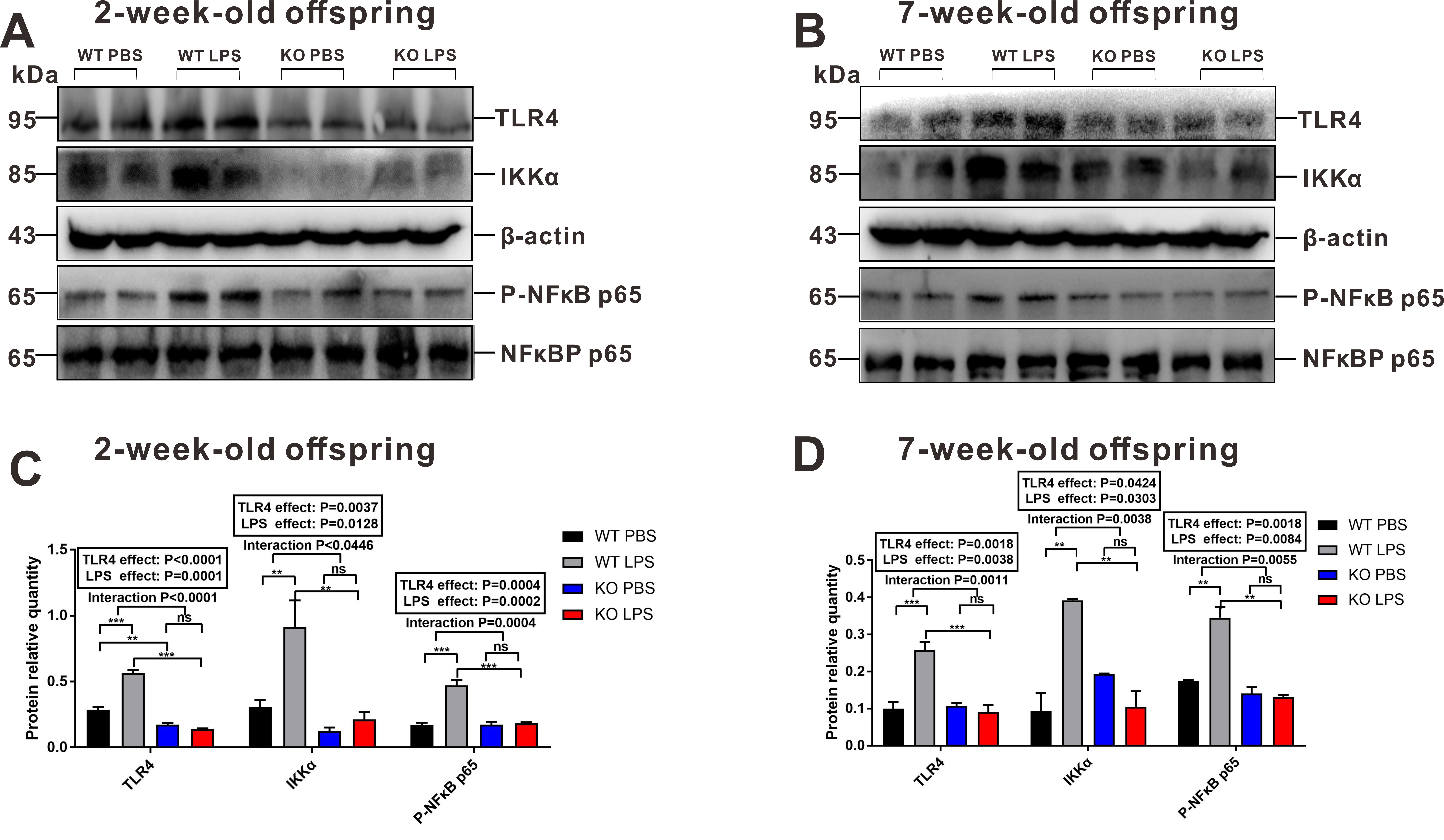
**

Figure 2. Comparison of the TLR4 signaling pathway-associated protein expression in the prefrontal cortex of two-week-old and seven-week-old offspring treated by PBS or LPS during the gestation period. The levels of TLR4, IKKα and P-NFκB 65 protein expression in the prefrontal cortex of (A) two-week-old and (B) seven-week-old offspring among the four groups. The quantification analysis of the level of TLR4, IKKα and Phospho-NFκB p65 protein expression in the prefrontal cortex of (C) two-week-old and (D) seven-week-old offspring among the four groups (n=3). The values are expressed as the means ± SEMs. “Interaction” indicates an effect of the LPS in the TLR4^-/-^ vs. WT mice; ns, not significant, ^*^*P* < 0.05; ^**^*P* < 0.01; and ^∗∗∗^*P* < 0.001.
